# Greenness and sustainability of common biosolvents in analytical chemistry

**DOI:** 10.1007/s00216-026-06559-2

**Published:** 2026-05-16

**Authors:** Elefteria Psillakis

**Affiliations:** Laboratory of Aquatic Chemistry, School of Chemical and Environmental Engineering, Technical University of Crete, Polytechnioupolis, 73100 Chania, Crete Greece

**Keywords:** Bio-based solvents, Green analytical chemistry, Sustainability, Life cycle thinking

## Abstract

Solvents derived from renewable biomass are often viewed as inherently greener and more sustainable alternatives to petrochemicals. However, their bio-origin does not always guarantee these attributes, as they can still be energy-intensive to produce, toxic, and poorly biodegradable, with enduring trade-offs related to land competition, resource use, and technological maturity. This contribution critically examines selected commercial biosolvents for which sufficient life cycle data are available and underscores the need to verify green claims on solvents through a life cycle approach. It further challenges the assumption that structurally identical bio-based solvents are inherently greener, demonstrating that upstream environmental impacts may still occur. Common neoteric solvents, such as ionic liquids and deep eutectic solvents, are also examined, highlighting the importance of measuring unresolved environmental and toxicological data. Furthermore, water, often proposed as a green solvent due to its abundance and ability to dissolve many substances, is discussed, with attention to the challenges associated with its disposal. Ultimately, this article is a call to fill the critical knowledge gap in life cycle solvent data and emphasizes that solvent greenness and sustainability cannot be presumed. Instead, they require a holistic approach that moves beyond reliance on simplified green narratives and integrates rigorous life cycle assessment, ethical feedstock sourcing, and economic viability.

**Graphical abstract:**

**Figure:**
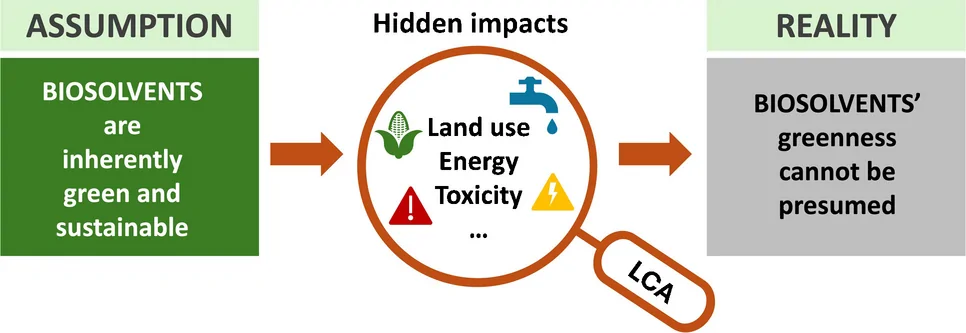

## Introduction

Solvents are indispensable, performance-defining components in analytical chemistry. Their use, however, is highly diffuse and spread across countless laboratories worldwide rather than concentrated within a finite number of large industrial facilities. Consequently, while each laboratory may be a minor point source, the sector collectively functions as a significant diffuse source of solvent waste. For example, reversed-phase high-performance liquid chromatography (RP-HPLC) can generate under typical operating conditions over a liter of waste per day [1, 2]. Although this volume may seem negligible in an industrial manufacturing context, the pervasive deployment of HPLC systems and the high sample throughput needs (e.g., large pharmaceutical companies) result in a significant cumulative environmental burden [1]. Traditional sample preparation techniques further illustrate this challenge. Liquid-liquid extraction (LLE) can consume hundreds of milliliters of halogenated solvents per batch, while solid-phase extraction (SPE) scales linearly solvent volume with sample throughput [3]. In both cases, the resulting waste stream often amounts to thousands of liters annually per laboratory.

The environmental, health, and safety (EHS) impacts of solvents have raised concerns, leading regulatory frameworks such as the European Union's REACH (Registration, Evaluation, Authorization, and Restriction of Chemicals) to strictly regulate solvents like benzene, toluene, dichloromethane, N,N-dimethylformamide, and chloroform [4]. To further mitigate solvent impacts, substantial ongoing efforts focus on minimizing their use (e.g., solventless microextraction, miniaturization) or replacing solvents of concern with greener alternatives [1, 5, 6]. In analytical chemistry, the identification of greener solvents typically prioritizes EHS criteria, but, in alignment with other disciplines should also involve a comprehensive life cycle approach that considers impacts from feedstock sourcing to waste management, including production energy demand, potential for recovery and end-of-life disposal [7–10]. This life cycle perspective helps analysts understand hidden costs, risks, and trade-offs, thereby avoiding the relocation of risks to another stage of the solvent’s life [7]. Under the aegis of green chemistry, Gu and Jerome [11] proposed 12 criteria that a green solvent needs to meet, and the authors concluded that a solvent fulfilling all 12 criteria unfortunately does not exist. Indeed, the concept of “green” solvent has been critically evaluated on several occasions, and a universal green solvent was suggested to be an unattainable ideal [10, 12].

The mounting EHS and economic concerns associated with traditional, fossil fuel-derived solvents have catalyzed the search for environmentally sustainable alternatives derived from renewable sources [13, 14]. In this context, bio-based solvents have gained significant attention as promising greener alternatives to traditional petroleum-derived products [15, 16]. This interest was primarily driven by the potential of renewably sourced ingredients to lower carbon footprint and to provide safer, more biodegradable, and less toxic solvents [11]. However, these attributes are not automatically guaranteed, and considering biosolvents ipso facto green is a specious assumption [17]. All too often, green claims on solvents rely on single-issue justifications, such as “it is green because it is biomass-derived” [12]. In reality, a biomass-derived solvent can still be energy-intensive to produce, toxic, and poorly biodegradable, highlighting the need to verify any green claims [17, 18]. Another critical misconception is to associate the bio-based origin of a solvent with inherent sustainability [19]. In a literal sense, bio-based solvents are sustainable because they are derived from renewable resources rather than finite geological reserves. However, true sustainability entails a broader approach that extends beyond a solvent’s EHS profile to include social and economic considerations, i.e., the three pillars of sustainability [19]. Consequently, solvent sustainability will depend on its “green” characteristics, but also on how (and indeed where) the solvent is to be sourced, managed and disposed of [10]. Economic factors should also be considered, as biomass-derived solvents are typically priced 15–30% higher than their petroleum-based counterparts [20]. Although increases in production scale and improvements in processing technologies are projected to gradually narrow this price gap in the coming years [20], the current economic barrier is particularly acute for developing countries, where the use of toxic solvents (e.g., dichloromethane) persists because of their low cost and availability [13].

This contribution critically examines common renewably sourced solvents for which sufficient environmental and toxicological life cycle data exist. Solvents discussed include biosolvents structurally equivalent to fossil-derived solvents, several neoteric solvents, and water. Highly promising solvents (e.g., supramolecular biosolvents (bioSURPAS)) are not included, as, to the best of the author’s knowledge, environmental and toxicological life cycle data are currently unavailable. Thus, the primary aim of this contribution is not to provide a comprehensive overview of the latest trends in biosolvents, their technical specifications, or emerging applications. Instead, the aim is to build upon available life cycle data on biosolvents and to highlight the often-overlooked life cycle impacts and costs. By applying the rigorous, life cycle–based lens of greenness and sustainability, this work calls for verifying green claims on solvents rather than presuming them, and for filling the gaps in current unresolved environmental, toxicological, and life cycle data.

## Biosolvents: pathways, feedstocks, and challenges

Bio-based solvents can be synthesized via several pathways, such as the direct fermentation of biomass, the chemical transformation of its derivatives, and the valorization of compounds or by-products from existing processes [21]. The most holistic approach, however, integrates these pathways within the biorefinery concept [22]. Analogous to a petroleum refinery, a biorefinery is a processing platform designed to convert biomass into a spectrum of individual marketable products, including platform chemicals that serve as precursors for both existing and new solvents [14, 22]. The latter is exemplified by the platform chemical levulinic acid, produced from the acid treatment of natural sugar sources (e.g., starch, cellulose, or cane sugar), which provides routes to a series of downstream products including 2-methyltetrahydrofuran [23].

Plant biomass is currently established as the primary source of renewable chemicals and solvents [13, 22]. Several biofuels (e.g., bioethanol) can also be used as renewable solvents [13], though additional purification steps are required to move from fuel-grade to solvent-grade products [24]. Biofuels are categorized by the generation of their feedstock, with the first generation (1G) being produced from edible biomass like sugarcane, corn, potatoes, cereals, and grains [13, 25]. The cultivation of these dedicated crops for biomass production can offer secondary benefits such as increased agricultural income, soil carbon sequestration, and improved air quality through enhanced plant cover [13]. However, while 1G pathways are technologically mature and offer favorable yields via relatively uncomplicated processes [26], their fundamental reliance on food resources places them in direct competition with global food and feed supply chains, raising critical concerns about resource allocation and land use change [25, 26]. To circumvent these concerns, research has pivoted towards second-generation (2G) feedstocks, using agricultural residues, grasses, and agroforestry products (lignocellulosic biomass) [13]. Despite technological advances, processing this feedstock remains energy-intensive, costly, and often results in lower yields compared to 1G pathways [26]. In response to these limitations, third-generation (3G) feedstocks, primarily derived from algae and cyanobacteria, and fourth-generation (4G), using genetic engineering to increase desired traits of organisms used in biofuel production [27], have emerged as promising alternatives, though their development remains largely confined to demonstration and pilot-scale projects [26].

The current production landscape is still dominated by 1G and 2G processes [26, 27] while the anticipated growth in bio-based chemicals production capacity (excluding bioethanol) has yet to materialize. The main reasons for this stagnation are threefold: first, the persistently high feedstock costs compared to oil prices, undermining the economic competitiveness of bio-based alternatives [28]; second, the fundamental mismatch in economies of scale between established fossil-based and emerging bio-based production capacities; and third, the lack of robust policy frameworks to facilitate the transition to a bio-based economy [29].

## Biosolvents structurally equivalent to fossil-derived solvents

**Fig. 1:**
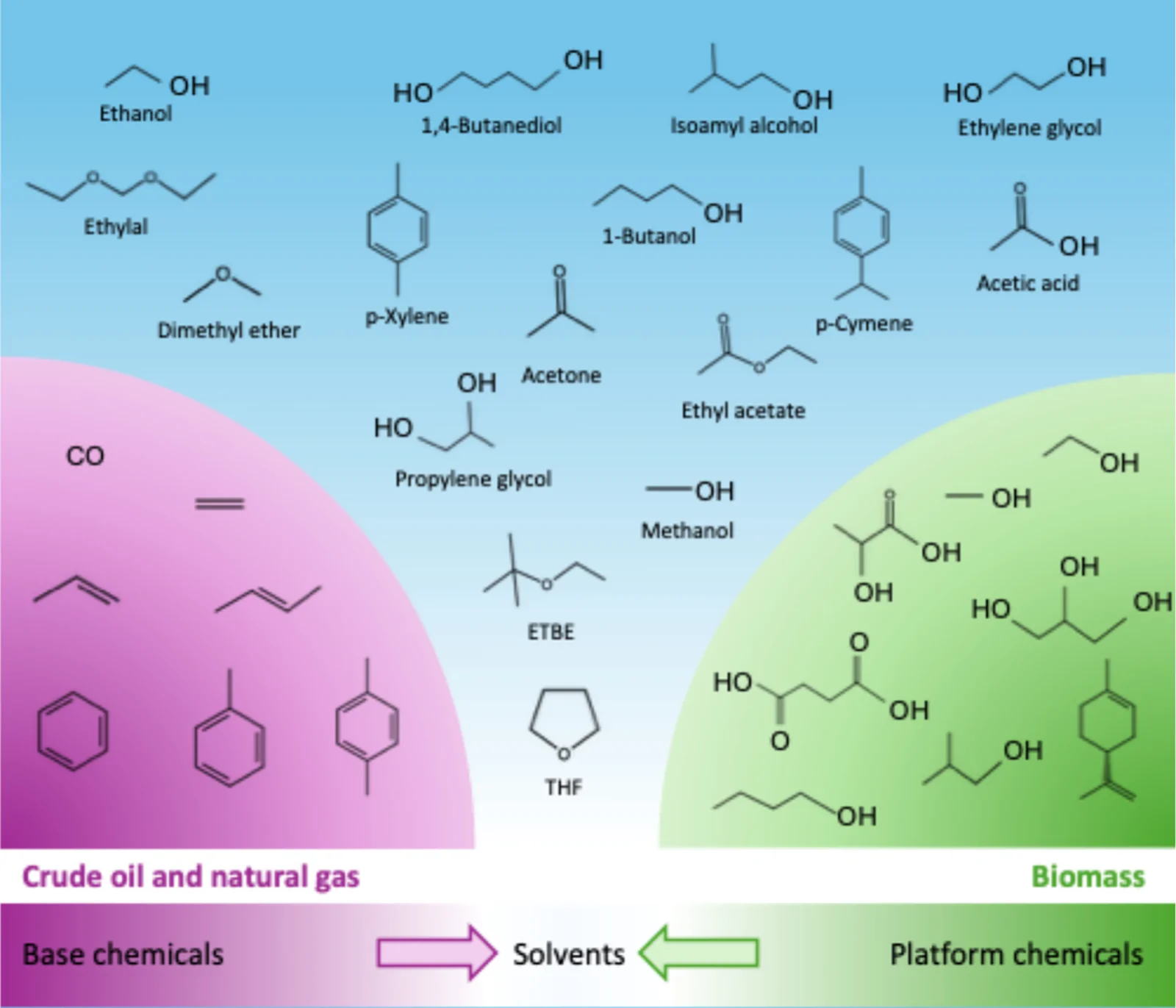
Solvents common to the petrochemical industry and the emerging bio-based economy. Adapted from Clark et al. [15]

Several bio-based solvents are structurally identical to those produced by the petrochemical industry (Fig. 1) [15] and a common misconception holds that they are inherently “greener” by virtue of their renewable origin [12]. However, upstream effects, e.g., increased land use and high energy demand for feedstock processing, may still occur during the production stage [19]. In contrast, fossil-derived solvent production relies on petrochemical pathways that have been refined and optimized at scale over decades, achieving energy efficiency and relatively low environmental emissions [7, 30]. A cradle-to-gate life cycle assessment (LCA) is therefore essential for a rigorous, comparative evaluation of the environmental impacts and energy requirements of both production routes [31].

Notwithstanding these concerns, these biosolvents are not a special case during the laboratory use and disposal stages and their EHS impacts are determined by their chemical structure, not their origin [18]. Thus, it should always be kept in mind that replacing a fossil-derived solvent with a structurally identical bio-based solvent will not mitigate the EHS risks of this solvent during laboratory use or waste disposal [14].

In general, assessing the sustainability of solvents introduces a layer of complexity and requires extending the evaluation beyond traditional EHS criteria to include geopolitical and ethical considerations related to feedstock sourcing. A prime example is bioethanol, the most mature and widely produced bio-based chemical [21, 29], also marketed as a solvent in analytical chemistry. Bioethanol is derived from diverse feedstocks such as maize, sugarcane, wheat, sugar beet, molasses, and lignocellulosic biomass [25, 32]. The environmental impact as well as resources and energy needed during production are variable and depend on the chemical composition of the feedstock as well as the efficiency of the pre-treatment technology used. For instance, LCA studies showed that bioethanol derived from rice straw has a higher water footprint and cumulative energy demand than ethanol produced from corn stover or cassava straw [33]. More importantly, while 1G bioethanol (derived from crops such as maize and sugarcane) may initially appear to offer a greenhouse gas emissions advantage over fossil-based ethanol, comprehensive LCA accounting for land use impacts has shown that fossil-based ethanol can instead be the preferable option [32, 34].

The scale of bioethanol production and its primary dependence on 1G feedstock raises serious concerns about its reliance on food crops and their by-products, particularly in the context of a growing global population [35]. The increasing bioethanol demand as a fuel exerts upward pressure on land use, potentially driving farmers to divert existing crops or croplands and plow up more forest and grassland [36]. This process, known as indirect land use change (ILUC), releases significant quantities of stored CO_2_, which in the case of biofuel production, can negate or even reverse its intended carbon savings [34]. It has been argued that ILUC is linked to increases in global food prices and food insecurity for vulnerable populations, while also promoting the creation of land consolidation and the exploitation of “marginal” land in developing countries [37].

## Neoteric solvents

Neoteric solvents are renewable alternatives that are structurally distinct from conventional petrochemical solvents [15, 38]. The term neoteric was proposed by Seddon in 1997 [39] to indicate “*a class of novel solvents that have remarkable new properties, that ‘break new ground’ and that offer a huge potential for industrial application.*” The use of neoteric solvents in analytical chemistry has been extensively reviewed in the past (e.g., [5, 9, 40, 41]), with the most common categories including, but not limited to, ionic liquids (ILs), deep eutectic solvents (DES), supercritical fluids, and bio-based solvents (such as 2-methyltetrahydrofuran, d-limonene, ethyl lactate, glycerol, and dihydrolevoglucosenone to name a few [13]).

**Fig. 2:**
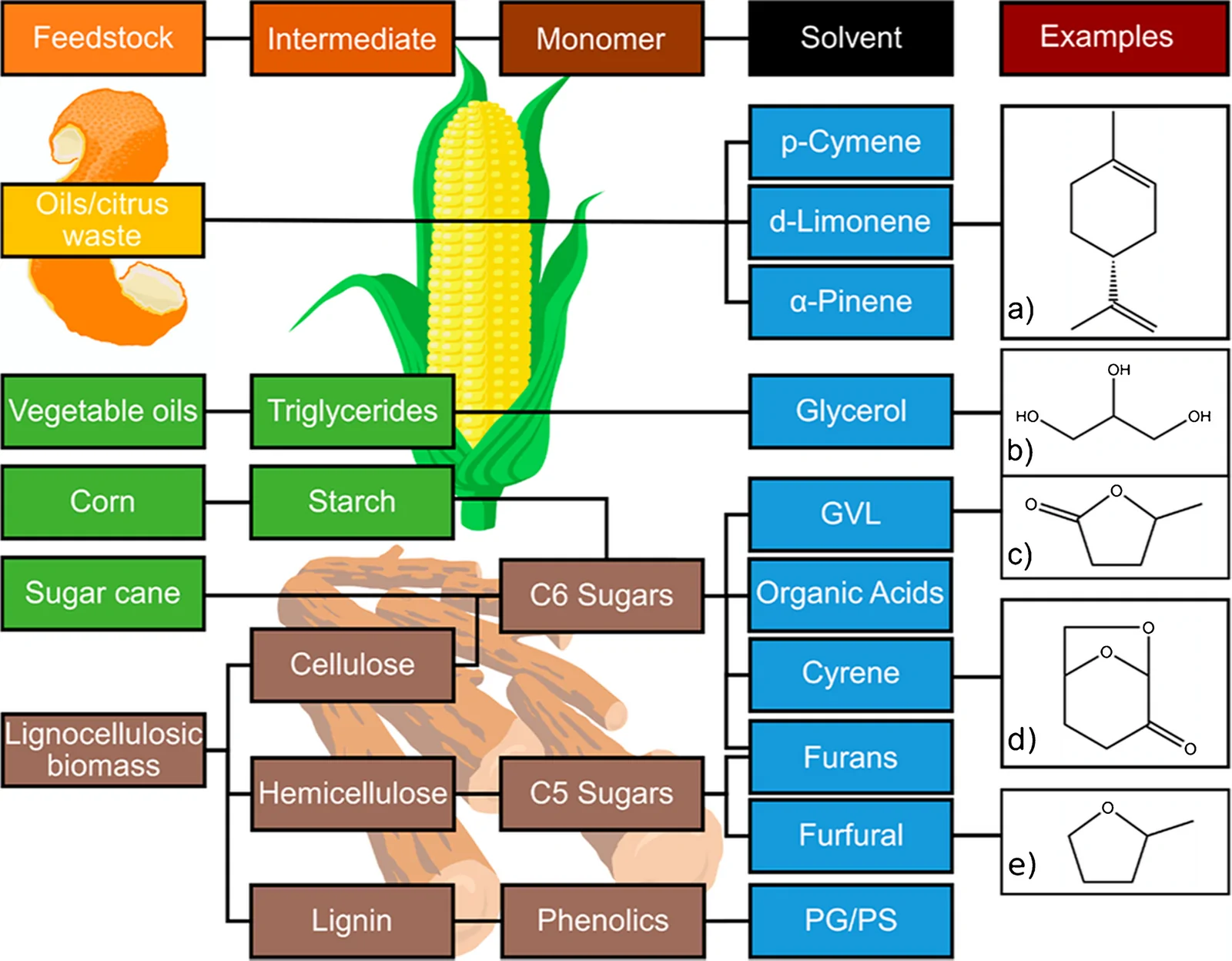
Examples of neoteric solvents derived from plant biomass. Structural examples: (**a**) d-limonene, (**b**) glycerol, (**c**) γ-valerolactone, (**d**) dihydrolevoglucosenone, and (**e**) 2-methyltetrahydrofuran. Taken from Clarke et al. [13] with permission

 In general, biomass as a feedstock is chemically distinct from the petroleum sources used for conventional solvents. As a result, many biomass-derived small molecules, whether serving as solvents themselves or as precursors, are not structural analogues of traditional petrochemical solvents (Fig. 2). For example, 2-methyltetrahydrofuran (2-MeTHF) is structurally distinct from both dichloromethane [42] and tetrahydrofuran, for which it has been proposed as a substitute [42, 43]. This structural divergence is also evident in dihydrolevoglucosenone (Cyrene™), which, despite lacking the nitrogen moiety, has been promoted as a replacement for toxic dipolar aprotic solvents, such as N-methyl-2-pyrrolidone in organic synthesis [15] and more recently, as an alternative to chromatographic solvents like acetonitrile [44]. Another bio-derived solvent is d-limonene, a monoterpene hydrocarbon extracted from citrus peel, that serves as a bio-based replacement for conventional solvents in multiple analytical applications, such as a substitute for toluene in moisture analysis and for n-hexane in the extraction of e.g., acidic drugs from aqueous samples [9, 11].

Neoteric solvents share several characteristic properties, such as low vapor pressure, high viscosity, thermal stability, and the ability to dissolve a wide range of substances [45]. However, these characteristics may present drawbacks, such as high viscosity that impedes mass transfer and handling [9, 13]. Among supercritical fluids, carbon dioxide is the most used, being of renewable origin, nontoxic, incombustible, easily available, and cheap. However, the use of supercritical fluids is constrained by the need for expensive high-pressure equipment and high operational energy [9].

Beyond these practical limitations lies a more fundamental concern: neoteric solvents are not inherently safer, less toxic, or more environmentally benign compared to conventional solvents. 2-MeTHF and Cyrene™ have been ranked as problematic in the CHEM21 selection guide, owing to health and safety concerns (2-MeTHF) or low environmental score (Cyrene™) [46]. It is noted that Cyrene™ was also linked to health hazards and, according to the Globally Harmonized System of Classification and Labelling of Chemicals (GHS) classification, causes skin and eye irritation and may also cause respiratory irritation [47]. At the same time, d-limonene is highly toxic towards aquatic organisms [9], costly, and may consume more energy than that when using hexane [11]. Another prominent example is ILs that were initially categorized as “green” based on their advantageous physicochemical properties (e.g., negligible volatility and low flammability) [8, 48]. However, this classification has since been challenged, with research findings indicating that ILs are not inherently more environmentally benign than the fossil-based solvents they were intended to replace [12]. Indeed, the initial belief that involatile solvents, such as ILs, are better than volatile ones had led in many cases to replacing one environmental impact with another. Current knowledge on ILs and their metabolites concludes that different IL families are eco(cyto)toxic, poorly biodegradable, and capable of forming harmful secondary metabolites [8, 49]. Furthermore, their synthesis can be very energy-intensive, at least at a laboratory scale, and rely on hazardous solvents and organic reagents [8, 12]. Therefore, the broad assumptions of health and safety based on renewable feedstocks, especially of low volatility, are scientifically challenged, and ongoing research is focused on better understanding their properties and elucidating their behavior [50].

In the case of DES, similar concerns apply and recent studies refuted the idea that DES are completely nontoxic in nature [51]. In fact, the biological effects of any DES are often found to be significantly different from those of its individual components due to synergistic effects. Moreover, a recent study using reline (a 1:2 molar mixture of choline chloride and urea) as a model DES, suggested that reline’s in vivo toxicity resulted from alkaline stress caused by thermally generated by-products. In contrast, freeze-drying effectively mitigated the toxic effect  [52]. The authors concluded that the safety of DES does not only depend on its composition, but possibly also on its preparation. Toxicity data were also recently measured for natural DES (NADES), consisting of mixtures of two or more natural components interacting with each other by hydrogen bonds to form a supramolecular mixture. The toxicity of oral administration of a phenolic NADES extract from green coffee beans was evaluated under a short-term condition, inducing mortality to 2 out of 12 rats [53]. This occurred even though this NADES extract contained polyphenols, whose beneficial effects have been demonstrated. In a more recent report, the in vitro assessment showed that NADES prepared with acids were more cytotoxic than NADES prepared with alcohols, an effect that was also confirmed by the in vivo assessment [54]. The authors also concluded that ecotoxicological assessment can help in the selection of component combinations for the design of safe NADES.

## Water as a green solvent

Water consistently occupies the top position in green solvent selection guides due to its inherent advantages of being abundant, non-toxic, safe to use, and inexpensive [7]. Beyond these foundational green credentials, water possesses unique physicochemical properties that make it indispensable as a diluent and a critical aqueous component of e.g., chromatographic mobile phases. The use of water as an extractant has also been explored in continuous and discontinuous formats, and the energy sources commonly used to accelerate the process from a variety of matrix types included heating, ultrasound, microwaves, and high pressure [55, 56].

The use of water is not only related to gains but also comes with often overlooked environmental costs. For example, based on a life cycle assessment (LCA) study, the energy demand was found to be a significant disadvantage for pressurized water extraction compared to supercritical fluid extraction in the recovery of antioxidants from rosemary leaves [5]. At the same time, the common perception of water as universally “green and safe” compared to organic solvents is misleading [57]. Water is only a truly green solvent if it can be directly discharged into and effectively treated by a wastewater treatment plant [58]. While pure water is environmentally benign, analytical workflows generate wastewater contaminated with various chemicals. Strict and costly regulations govern wastewater discharge, and effective treatment prior to safe release is not universally guaranteed [59]. This creates a major post-use challenge in terms of energy and infrastructure requirements that solvent selection guides praising its biodegradability do not account for [7].

## Outlook

The replacement of petrochemical solvents with bio-based alternatives based solely on the renewable origin of the latter is an inadequate strategy for addressing the adverse impacts of solvent use. A bio-based solvent is not automatically green, and any green claims require thorough verification as these solvents may still be energy-intensive to produce, toxic, or poorly biodegradable. Equally problematic is the assumption that bio-based origin equates to inherent sustainability. True “sustainability” is a broader approach that should include social and economic considerations next to EHS hazards. To begin with, the sustainability of the biomass, feedstocks must be questioned [18]. This is a pressing concern similar to that seen in bio-based fuels, where bioenergy crops have displaced food crops and caused deforestation raising concerns over global biodiversity [18]. Economic factors should also be considered as bio-based solvents remain more expensive to produce than their petrochemical counterparts. Despite promising developments, the small production scale for the majority of common biomass-derived solvents results in higher production costs compared to their petroleum-based counterparts. Compounding this issue, feedstock prices and availability fluctuate based on growing conditions, harvest timing, and competing uses (such as food and biofuel production), introducing supply chain vulnerabilities absent from fossil-derived supply chains [20].

Admittedly, fossil-derived solvents are not sustainable in the long term, and their replacement must be accelerated. However, this imperative does not justify the use of resources that compete with other supply chains or that may cause adverse impacts at other stages of a solvent’s life cycle. Nor can it justify the adoption of complex, energy-demanding, and wasteful synthesis routes [17]. The promise of a solvent derived from renewable biomass is frequently undermined by unexamined life cycle impacts, including indirect land use change, energy-intensive processing, and unresolved EHS hazards that persist during laboratory use and disposal phases. It is interesting to note that Clarke et al. [13] highlighted that it is entirely possible that using a small amount of an easily separated “traditional” organic solvent could provide a more sustainable process than e.g., a neoteric solvent. It is therefore necessary to conduct a full analysis of the entire life cycle for any solvent, before they can be classified as green. As noted in the literature, environmental benefits are not “automatic”. While certain bio-based solvents demonstrate clear advantages, the greenness and sustainability of others is highly contingent on the feedstock and production pathway [5]. Furthermore, the purification of bio-based solvents often requires energy-intensive steps like distillation or dehydration, which can offset their initial green benefits and add to the overall process complexity [5, 11]. Consequently, the development of new and promising biosolvents must be accompanied by studies that address not only their environmental and toxicological data but also their impact from a life cycle perspective.

Commercially available biosolvents, such as methanol, ethanol, and acetonitrile for HPLC, are now a reality in analytical chemistry, and their market promotion often focuses on official test methods like ASTM D6866 to quantify their biogenic carbon content [18]. However, critical information such as feedstock generation, agricultural practices, geographic origin, or production energy requirements is not visible, creating a significant feedstock transparency gap. Procurement policies must demand full feedstock transparency and favor products with credible sustainability certifications. Furthermore, method development should intrinsically incorporate green analytical chemistry principles, evaluating not only a biosolvent’s performance but also the cumulative impact of its production and end-of-life treatment. Analytical chemists must become discerning consumers, critically evaluating marketing claims in which terms such as “bio-based” may function as opaque marketing statements rather than verifiable life cycle attributes.

The path forward requires a fundamental shift from seeking solvent replacements based on bio-origin to making informed decisions in solvent selection from a life cycle perspective by evaluating green credentials, navigating complex trade-offs, and managing hidden costs. Along this path, it should always be remembered that “the greenest solvent is the one that is not used”. Thus, the practical goal is less about pursuing a “mythical” green and sustainable biosolvent and more about making informed choices that decrease solvent use and minimize overall impact.
